# Healthy Lifestyle Does Matter for Community-Dwelling Adults Pursuing Quality of Life

**DOI:** 10.1093/geroni/igab046.1347

**Published:** 2021-12-17

**Authors:** Li-Fan Liu

**Affiliations:** National Cheng Kung University, National Cheng Kung University, Tainan, Taiwan (Republic of China)

## Abstract

It has been well documented that socioeconomic factors influence lifestyle behaviors and all the physical and mental status at the individual level do matters for elderly people experiencing healthy aging. This study aimed to explore to what extent the healthy lifestyle including exercise and social participation influence on the health status of the community dwellings and their quality of life in Taiwan. Using a cross-sectional survey design, 1032 adults, aged ≥ 50 years, were interviewed with complete data from four communities in southern Taiwan. The results showed that for older community dwellings adults, doing exercise was significantly associated with feeling less stress, less depress, higher life satisfaction and higher quality of life (p<0.001). Being volunteers was found to be significantly associated with better quality of life (p<0.001). In the aging society, it is necessary to apply multifaceted approaches extending from individual solutions to public policy efforts in promoting healthier lifestyles.

